# Supplementary material for: MOF-808 as a Highly Active Catalyst for the Diastereoselective Reduction of Substituted Cyclohexanones
Source: Molecules. 2022 Sep 25;27(19):6315. doi: 10.3390/molecules27196315 (PMC9571754; doi:10.3390/molecules27196315)
Supplement: Supplementary file 1 [file molecules-27-06315-s001.zip › molecules-1860039-supplementary.pdf]

SUPPLEMENTARY MATERIALS

MOF-808 as a highly active catalyst for the diastereoselective reduction of substituted cyclohexanones

H. H. Mautschke and F. X. Llabrés i Xamena

Structure and characterization of MOF-808

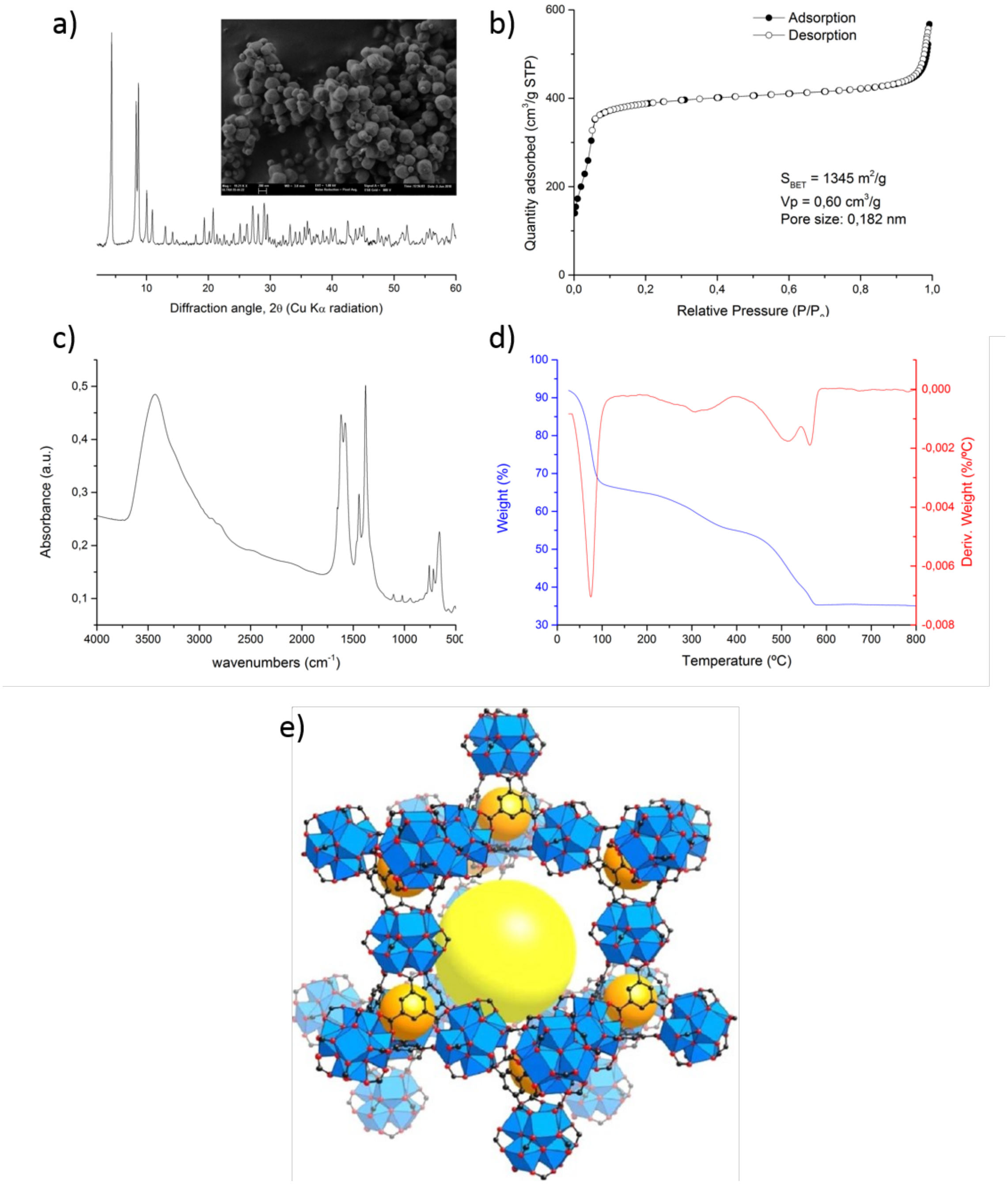

Figure S1. Characterization of MOF-808. a) XRD powder diffraction pattern (Cu Kα radiation). The inset shows the corresponding FESEM image, in which the crystal size (*ca.* 200 nm) and the octahedral-shaped morphology is evidenced. b) N_2 adsorption/desorption isotherm (at 77K) and corresponding textural parameters. c) FTIR spectrum. The absorption bands observed are analogous to those previously described for MOF-808 (see for instance: J. Xu et al., *New J. Chem.* **2019**, *43*, 4092-4099). d) TGA and DTG curves. e) Structure of MOF-808. Reproduced with permission from Furukawa et al. *J. Am. Chem. Soc.* **2014**, *136*, 4369–4381. Copyright 2014 American Chemical Society.

Synthesis of other non-commercial catalysts used in the work

Zr-beta. Preparation of this catalyst consisted of two steps:

a) Synthesis of dealuminated Zr-beta seeds

1.85 g of $AlCl_3 \cdot 6H_2O$ were dissolved in 4.33 g of water. To this solution, 45.2 g of tetraethylammonium hydroxide solution (TEAOH, 35 wt. % in H_2O) were added, followed by 40.0 g of tetraethyl orthosilicate (TEOS), and the mixture was stirred until evaporation of the ethanol formed by hydrolysis of TEOS. The final composition of the gel was:

SiO_2: 0.56 TEAOH: 0.02 Al_2O_3: 6.5 H_2O

The gel was then transferred into a teflon lined autoclave and heated to 140ºC for 3 days with stirring. The solid product was recovered by filtration, washed with distilled water and dried at 100ºC. The resulting zeolite was dealuminated by treating 1 g of the solid with 60 g of HNO_3 (60 wt%) at 80ºC for 24 h. The dealuminated solid was filtered, washed with water and dried at 100ºC. The final Si/Al ratio of the solid was higher than 2000, as determined by elemental analysis.

b) Synthesis of aluminum-free Zr-beta zeolite

30 g of TEOS and 33.0 gr of TEAOH (35 wt. % in H_2O) were placed inside a teflon lined autoclave. To this, a solution containing 0.39 g of $ZrOCl_2 \cdot 8H_2O$ in 2.75 g water was added, and the mixture was stirred until evaporation of the ethanol formed by hydrolysis of TEOS. To this solution, 3.27 g of HF (48 wt%) were added, and the resulting viscous gel was mixed with a aqueous suspension of 0.36 g of preformed dealuminated Zr-beta seeds in 2 g of water. The final composition of the gel was:

SiO_2: 0.54 TEAOH: 0.008 ZrO_2: 0.54 HF: 7.5 H_2O

The gel was then heated to 140ºC for 14 days with stirring. The solid product was recovered by filtration, washed with distilled water and dried at 100ºC. The final Si/Zr ratio of the solid was 148, as determined by elemental analysis.

Reusability of MOF-808

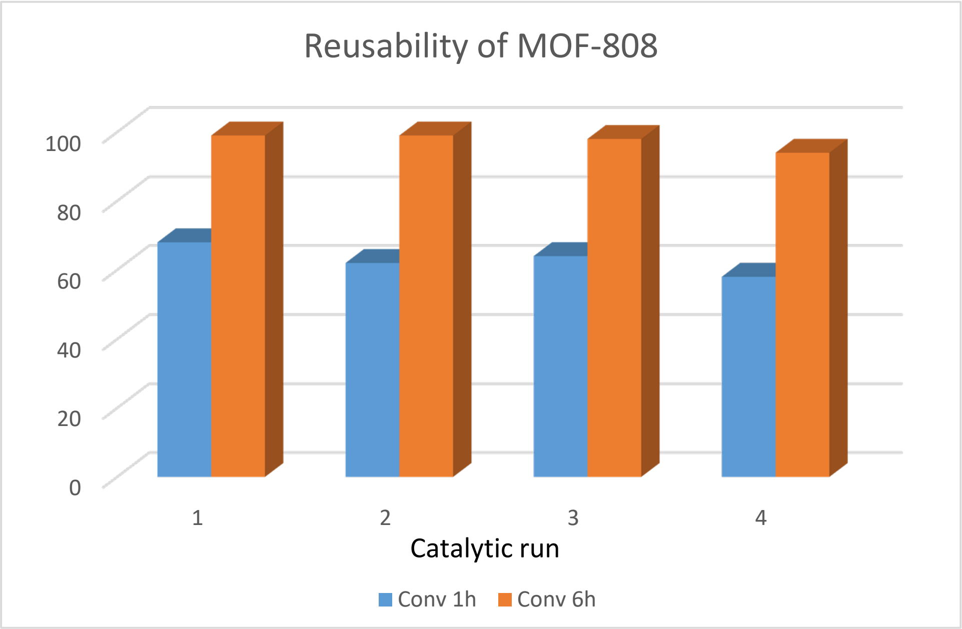

Figure S2. Conversion of 3MeCH=O obtained (after 1 and 6 h of reaction) after 4 consecutive cycles over MOF-808 using 2-BuOH as solvent at 80 ºC.

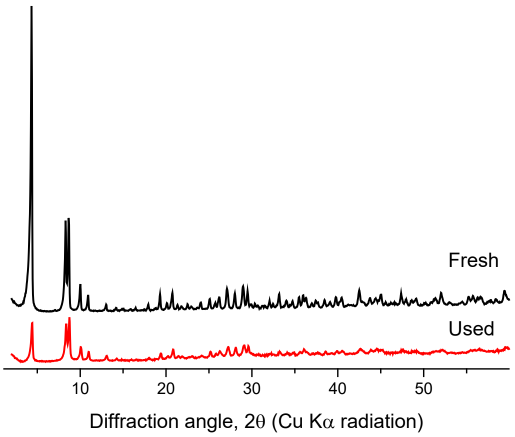

Figure S3. XRD powder diffraction pattern (Cu Kα radiation) of fresh MOF-808 and after four catalytic cycles of 3MeCH=O reduction.
